# Inhibition of protein phosphatases attenuates A_1_-adenosine receptor-stimulation induced negative inotropic effects of cAMP-increasing agents in the isolated human atrium

**DOI:** 10.1007/s00210-025-03854-0

**Published:** 2025-02-05

**Authors:** Rebecca Schwarz, Britt Hofmann, Ulrich Gergs, Joachim Neumann

**Affiliations:** 1 Institute for Pharmacology and Toxicology, Medical Faculty, Martin-Luther-University Halle-Wittenberg, Magdeburger Str. 4, 06097 Halle (Saale), Germany; 2 Cardiac Surgery, Medical Faculty, Martin-Luther-University Halle-Wittenberg, Ernst Grube Str. 40, 06097 Halle (Saale), Germany

**Keywords:** Cantharidin, Adenosine receptor, Human atrium, Phosphatases, CAMP

## Abstract

N^6^-(R)-Phenylisopropyladenosine (R-PIA), an agonist at A_1_-adenosine receptors, alone exerts negative inotropic effects (NIE) in the human atrium. This NIE is augmented in the presence of cAMP-increasing agonists like phosphodiesterase inhibitors (cilostamide, rolipram) or a direct activator of adenylyl cyclase (forskolin). Cantharidin inhibits protein phosphatases 1 and 2A (PP1, PP2A). We hypothesized that cantharidin would attenuate this NIE of R-PIA in the presence of cilostamide or forskolin. During open heart surgery (patients were suffering from severe coronary heart disease), isolated human atrial preparations (HAP) were obtained. These HAP were mounted in organ baths and electrically stimulated (1 Hz). For comparison, we studied isolated electrically stimulated (1 Hz) left atrial preparations (LA) from wild type mice. We noted that R-PIA exerted negative inotropic effects in LA and HAP in the presence of cilostamide or rolipram and forskolin that were attenuated by cantharidin. We hypothesize that R-PIA in the presence of phosphodiesterase inhibitors or forskolin stimulates PP in the human atrium. Hence, R-PIA acts, at least in part, by stimulating PP in HAP.

## Introduction

**Fig. 1:**
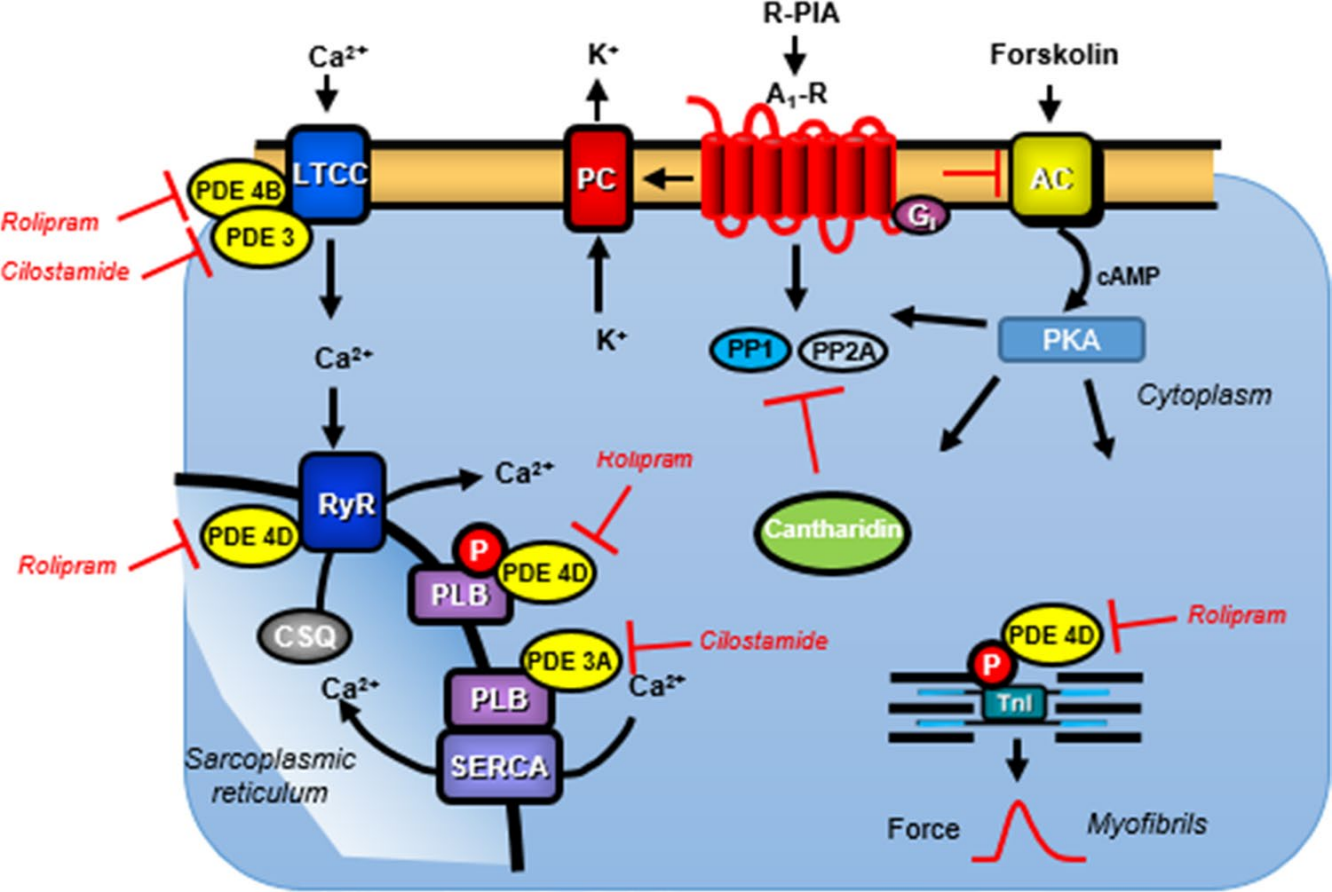
Phosphodiesterase (PDE) isoenzyme inhibition (by rolipram or cilostamide) and forskolin increase cAMP levels in the human atrium are depicted. This cAMP activates cAMP-dependent protein kinases (PKA). PKA then phosphorylates regulatory proteins in the human atrium. Cardiac relaxation is brought about by phosphorylation of phospholamban (PLB). Cardiac contraction is in part mediated by ryanodine receptors (RyR). The activities of protein phosphatases (PP) PP1 and PP2A are inhibited by cantharidin. R-PIA via stimulation of A_1_-adenosine receptors may inhibit the enzymatic activity of adenylyl cyclase (AC) via a pertussis-toxin-sensitive G-protein (G_i_) and may open potassium channels (PC) in the sarcolemma or may close L-type calcium ion (Ca^2+^) channels (LTCC) and may directly or indirectly activate PP

Β-adrenoceptors activate adenylyl cyclases via stimulatory guanosine-triphosphate (GTP)-binding proteins and lead thereby to the formation of 3′,5′ -cyclic adenosine monophosphate (cAMP) in the human heart (Fig. 1). Thereafter, in the myocardium, cAMP activates kinases (PKA) that phosphorylate and thereby activate several regulatory proteins. These phosphorylations are reversed by PP (Herzig and Neumann 2000, Neumann et al. 2021a).

We have demonstrated in guinea pig and human preparations that cantharidin inhibited PP1 and PP2A from hearts (Neumann et al. 1995a, b). Cantharidin increased the force of contraction in guinea pig papillary muscle and in human atrial and ventricular preparations via increasing the phosphorylation state of regulatory proteins (Neumann et al. 1995b; Schwarz et al. 2023a). The positive inotropic effect of isoprenaline can be attenuated in the ventricle but also in the atrium by A_1_-adenosine receptor stimulation, typically by R-PIA. This has been shown in guinea pig hearts, mouse hearts, human atrium, or human ventricle (Böhm et al. 1984, Böhm et al. 1985a,b, Böhm et al. 1988, Böhm et al. 1989, Boknik et al. 2001, Boknik et al. 2009, Schwarz et al. 2023b). A similar observation has been reported for acetylcholine. Acetylcholine per se reduces the FOC in the mammalian atrium (in vitro or in vivo) but this effect is amplified in the presence of isoprenaline or more generally when the sympathetic nerve system is activated: this was called accentuated antagonism (Levy 1971).

Moreover, cAMP levels can be increased independently of sarcolemmal β-adrenoceptors by direct stimulation of the adenylyl cyclases. An example of this option is forskolin (Seamon and Daly 1981). Forskolin independently of sarcolemmal receptors can activate cardiac adenylyl cyclases and this will produce cAMP and increase the phosphorylation state of phospholamban and increase the force of contraction in guinea pig ventricles, HAP, and human ventricular preparations (Bristow et al. 1984, Lindemann and Watanabe 1985, Näbauer et al. 1988, Neumann et al. 1999a; Christ et al. 2014). The PIE of forskolin is attenuated by R-PIA, e.g., in isolated perfused guinea pig hearts (West et al. 1986).

If one assumes that R-PIA only reduces theforce of contraction by diminishing the activity of adenylyl cyclases via inhibitory G-proteins (Fig. 1) then R-PIA should not reduce the force of contraction that is augmented independently of any cAMP increase that occurs beyond an activation of adenylyl cyclases. However, this was reported in the HAP. Indeed, we recently published R-PIA can still decrease FOC if we gave dibutyryl-cAMP (Schwarz et al. 2024). This argues that PIA might not or not solely act via reducing cAMP production through adenylyl cyclases.

Moreover, cAMP is degraded by phosphodiesterases (PDE). In the mouse heart, PDE IV is mainly important and is inhibited by rolipram (Movsesian and Kukreja 2011; Neumann et al. 2019; Rayo Abella et al. 2023a, Fu et al. 2024). In the human heart, the PDE III is most important and is inhibited by cilostamide (Christ et al. 2006, Rayo Abella et al. 2023b). In the past, several studies used IBMX, a drug that inhibits several PDEs including PDE III and PDE IV (Movsesian and Kukreja 2011). Phosphodiesterase inhibitors increase the phosphorylation state of cardiac regulatory proteins in the human ventricle (Bartel et al. 1996) but also in the human atrium (Rayo Abella et al. 2023b). It was shown that IBMX increased FOC in human ventricular muscle strips (Näbauer et al. 1988, Steinfath et al. 1992). R-PIA reduced IBMX-stimulated FOC without reducing cAMP levels (guinea pig papillary muscles: Böhm et al. 1986). This would argue even more that cAMP reduction is not the cause of the reduction of cAMP-induced increases in FOC by R-PIA in the human atrium.

We have recently shown that the negative inotropic effect (NIE) of PIA alone (in the absence of cAMP-increasing drugs) was attenuated by cantharidin (Schwarz et al. 2024). We suggested this might be indirect evidence that the A_1_-adenosine receptor can activate PP. This seems not to be a unique phenomenon but a more generalized mechanism. Indeed, we also reported that M_2_-muscarinic receptor stimulation in the HAP might activate PP (Schwarz et al. 2023b).

Moreover, we and others have supplied evidence that the effect of R-PIA to reduce the force of contraction in the presence of β-adrenoceptor stimulation in the mammalian ventricle involves not inhibition of cAMP-production but activation of cardiac phosphatases (guinea pig: Gupta et al. 1998; Herzig et al. 1995).

Hence, we hypothesized that R-PIA might reduce FOC previously raised by β-adrenoceptor independent elevation of cAMP in the HAP by activating PP. Thus, we hypothesize that the NIE of R-PIA in the presence of forskolin or cilostamide in HAP is attenuated by cantharidin. As a confirmatory study, we tested the same hypothesis in the LA where we used also forskolin and rolipram (instead of cilostamide). The latter was done because cilostamide inhibits PDE III which is not important in the mouse heart while rolipram inhibits PDE IV which appears to be the main PDE in the mouse heart.

In brief, we formulated the main hypothesis:

Cantharidin can attenuate the R-PIA induced NIE in the presence of forskolin or cilostamide in HAP.

## Materials and methods

### Contractile studies in mice

In brief, the right or left atrial preparations from adult CD1 mice of random gender, were isolated and mounted in organ baths as previously described (Gergs et al. 2024; Neumann et al. 2003). The bathing solution of the organ baths contained 119.8 mM NaCI, 5.4 mM KCI, 1.8 mM CaCl_2_, 1.05 mM MgCl_2_, 0.42 mM NaH_2_PO_4_, 22.6 mM NaHCO_3_, 0.05 mM Na_2_EDTA, 0.28 mM ascorbic acid, and 5.05 mM glucose. The solution was continuously gassed with 95% O_2_ and 5% CO_2_ and maintained at 37 °C and pH 7.4 (Neumann et al. 1994, 2019). Spontaneously beating right atrial preparations from mice were used to study any chronotropic effects. The drug application was as follows. After equilibration was reached, cantharidin (100 µM) was added to left atrial or right atrial preparations. Then, where indicated, R-PIA was cumulatively applied to the preparations.

### Contractile studies on human preparations

The contractile studies on human preparations were done using the same setup and buffer as used in the mouse studies. The samples were obtained from the patients given in Table 1. Drug therapy is listed in Table 1. Our methods used for atrial contraction studies in human samples have been previously published and were not altered in this study (Rayo Abella et al. 2023b). Contracting human muscle strips were washed at least three times with 10 ml buffer in order to remove as far as possible any drug taken prior to surgery which might have interfered with our contraction measurements.

**Table 1:** Samples obtained from the patients and their drug therapy

Patient	Gender	Age in years	Disease	Drug treatment
#1	m	71	3-vessel CAD, Afib, IDDM II, status post	Apixiban, atorvastin, valsartan, metformin, bisoprolol, eplerenone, ezetimibe, empagliflozin, saxagliptin, pantoprazole, basal insulin
			DVT right, NYHA II, CCS III, EF 38%	
#2	m	47	ACS with concomitant insufficiency, NIDDM II, dyslipidemia, postherpetic neuralgia, status post alcohol abuse, severe nosocomial pneumonia, AH, NYHA III, CCS I, EF 25%	Amlodipine, atorvastatin, Entresto, eplerenone, empagliflozin, Nebivolol, pantoprazole, metformin, bisoprolol
#3	m	69	2-vessel CAD, AH, DVT right, HLP, prediabetes, NYHA I, CCS II-III, EF 68%	Candesartan, amlodipine, bisoprolol, atorvastatin, apixiban, ezetimibe, dapagliflozin, torasemide, pantoprazol, hydrochlorothiazide, clopidogrel
#4	f	59	1-vessel CAD, COPD, AVS in bicuspid aortic valve, Spinal stenosis, NYHA III-IV, CCS I-II, EF 60%	Atorvastatin, budesonide, lisinopril, hydrochlorothiazide, torasemide, pantoprazole, theophylline
#5	m	81	3-vessel CAD, AH, AVS, D.m. II, hypercholesterolemia, NYHA III, CCS III, EF 50%	Acetylsalicylic acid, empagliflozin, metformin, ezetimibe, atrovastatin, duloxetin, captopril, metamizole, amlodipine
#6	m	57	3-vessel CAD, AH, Prediabetes, HLP, status post STEMI, NYHA II, CCS II-III, EF 60%	Acetylsalicylic acid, atorvastatin, prasugrel, ramipril, ezetimibe, pantoprazol, bisoprolol
#7	f	69	2-vessel CAD, AH, HLP, severe AVS, exocrine pancreatic insufficiency, nicotine abuse, NYHA III, CCS II-III, EF 66%	Amlodipine, atorvastatin, bisoprolol, bromelain, ezetimibe, pantoprazol, torasemide, apixiban
#8	m	64	3-vessel CAD, AH, HLP, CKD III, chronic nicotine abuse, NYHA III, CCS III-IV, EF 50%	Acetylsalicylic acid, atorvastatin, bisoprolol, torasemide, pantoprazol, pirenatide
#9	m	72	2-vessel CAD, AH, HLP, severe AVS, ICM, paroxysmal VHF, NYHA III, CCS III, EF 55%	Amlodipine, atorvastatin, bisoprolol, torasemide, ezetimibe, apixiban, clopidogrel, tamsolusin, pantoprazole
#10	m	33	Ascending aortic aneurysm, severe AVR, NYHA III-IV, CCS I, EF 37%	Bisoprolol, torasemide, phenprocoumon, pantoprazol
#11	f	61	3-vessel CAD, HLP, AH, Nicotine abuse, NYHA II-III, CCS III, EF 40%	Acetylsalicylic acid, amlodipine, valsartan, ezetimibe, rosuvastatin

*Abbreviations*: *CAD* coronary artery disease, *HLP* hyperlipidaemia, *CCS* Canadian Cardiovascular Society, *EF* ejection fraction, *NYHA* New York heart disease, *Afib* atrial fibrillation, *NIDDM* non-insulin dependent diabetes mellitus, *ACS* acute coronary syndrome, *DVT* deep vein thrombosis, *AH* arterial hypertension: *STEMI* ST elevation myocardial infarction, *CKD* chronic kidney disease, *AVS* aortic valve stenosis, *ICM* ischemic cardiomyopathy, *m* male, *f* female. Age: age of patient on the day of cardiac surgery

### Data analysis

Data shown are means ± standard error of the mean. Statistical significance was estimated using the analysis of variance followed by Bonferroni’s *t*-test. A *p*-value < 0.05 was considered to be significant.

### Drugs and materials

The drugs cantharidin (CANT, stock solution 100 mM in dissolved dimethylsulfoxide (DMSO)), rolipram, cilostamide, forskolin, and R-PIA were purchased from Sigma-Aldrich (Germany). All other chemicals were of the highest purity grade commercially available. Deionized water was used throughout the experiments. Stock solutions were prepared fresh daily.

## Results

### Mouse atrium: rolipram

**Fig. 2:**
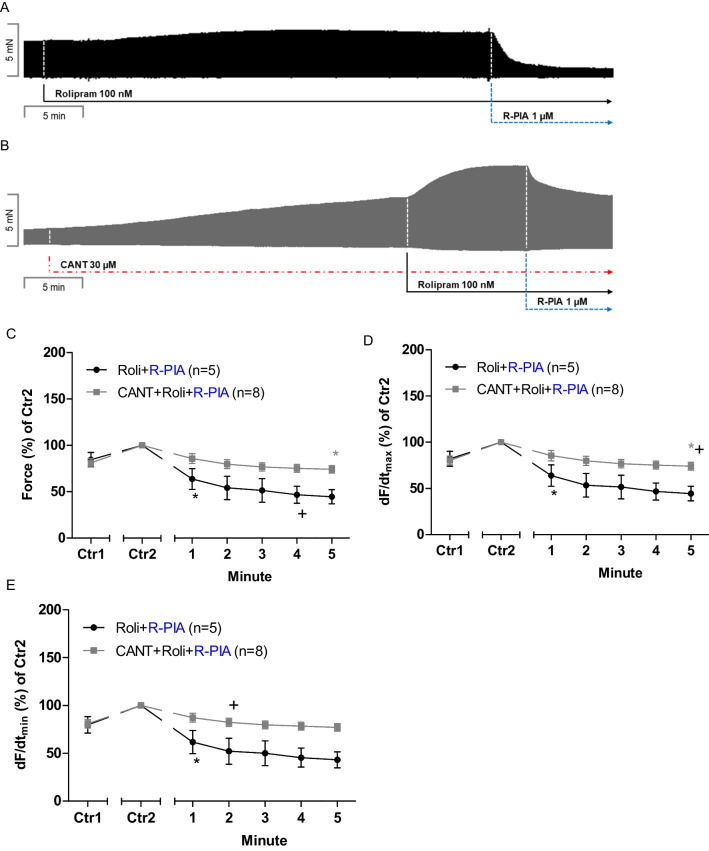
Effects of R-PIA in the presence of rolipram on contractile parameters in left and right mouse atrium in the presence or absence of cantharidin. **A** Original recording of the effect of R-PIA in the presence of rolipram on the force of contraction in milli Newton (mN, Ordinate) over time in minutes (min, Abscissa). **B** Original recording of the effect of R-PIA in the presence of rolipram on the force of contraction in the additional presence of 30 µM cantharidin (CANT) in milli Newton (mN, Ordinate) over time in minutes (min, Abscissa). **C** Diagram for the negative inotropic effect of R-PIA in the presence of rolipram alone or the additional presence of cantharidin (30 µM) in isolated electrically driven mouse left atrial preparations. Ordinates and abscissa give the force of contraction in % of pre-drug value or applied concentration of R-PIA, respectively. The asterisk (*) and plus sign ( +) indicate the first significant difference versus 30 µM cantharidin or time-matched control values (Ctr2) or R-PIA in the presence of cantharidin, respectively. Number (*n*) indicates the number of experiments. Ctr1 indicates pre-drug value. Ctr2 (100%) indicates plus/minus cantharidin. **D** Line diagram of the effects of R-PIA in the presence of rolipram or in the additional presence of cantharidin (30 µM) on rate of tension development in isolated electrically driven mouse left atrial preparations. Ordinate and abscissa give the rate of tension development (dF/dt_max_) in % of pre-drug value or applied concentration of R-PIA, respectively. The asterisk (*) and plus sign ( +) indicate the first significant difference versus 30 µM cantharidin or time-matched control values (Ctr2) or R-PIA in the presence of cantharidin, respectively. Number (*n*) indicates the number of experiments. Ctr1 indicates pre-drug value. Ctr2 (100%) indicates plus/minus cantharidin. **E** Line diagram of the effects of R-PIA in the presence of rolipram or in the additional presence of cantharidin (30 µM) on rate of tension relaxation in isolated electrically driven mouse left atrial preparations. Ordinate and abscissa give the rate of tension relaxation (dF/dt_min_) in % of pre-drug value or applied concentration of R-PIA, respectively. The asterisk (*) and plus sign ( +) indicate the first significant difference versus 30 µM cantharidin or time-matched control values (Ctr2) or R-PIA in the presence of cantharidin, respectively. Number (*n*) indicates the number of experiments. Ctr1 indicates pre-drug value. Ctr2 (100%) indicates plus/minus cantharidin

First, 100 nM rolipram was given to increase FOC. This concentration of rolipram stems from our previous studies in LA (Neumann et al. 2019). R-PIA (1 µM, Schwarz et al. 2024) was applied in the absence of cantharidin after the addition of rolipram (original tracing: Fig. 2A) or in the additional presence of cantharidin (Fig. 2B). In the presence of cantharidin, the negative inotropic effect of R-PIA is attenuated and the fall in force developed slower (original recordings in Fig. 2A and B). These data are summarized for the force of contraction in Fig. 2C. Note that Ctr1 indicates the developed tension in the absence of solvent or R-PIA (Fig. 2C). We define Ctr2 as the force noticed after 30 min of initial incubation with 30 µM cantharidin or solvent control and rolipram just before R-PIA was added to the organ bath. More results were noted for additional muscle parameters. When calculating the first derivate of force versus time, one notices that the rate of tension development was enhanced by cantharidin and rolipram, but additional R-PIA was more efficient to reduce this parameter in the absence than in the presence of cantharidin (Fig. 2D). Likewise, the rate of tension relaxation was enhanced by cantharidin in the presence of rolipram, but additional R-PIA was more efficient to reduce this parameter in the absence than in the presence of cantharidin (Fig. 2E).

### Mouse atrium: forskolin

**Fig. 3:**
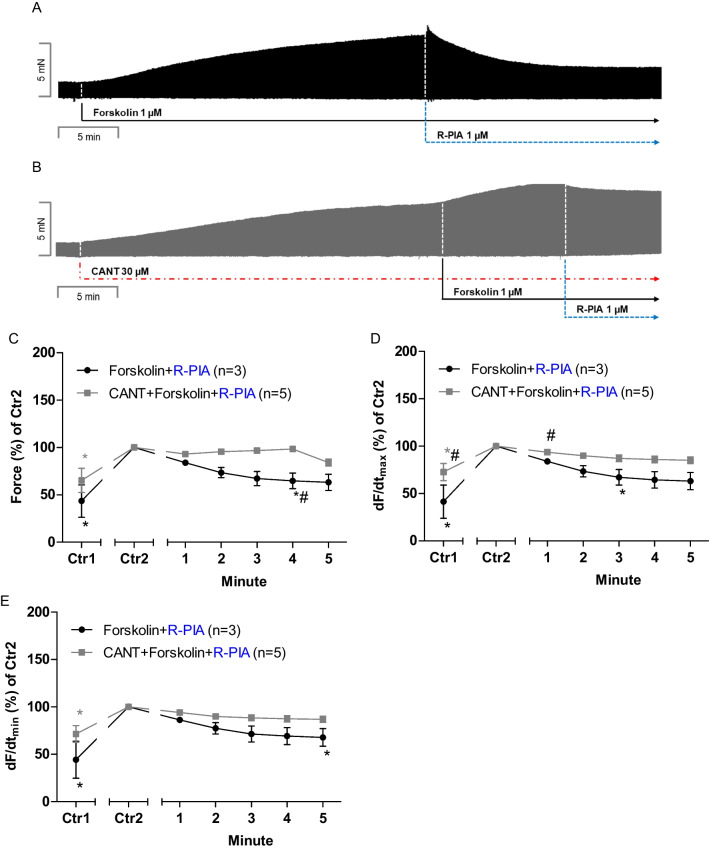
Effects of R-PIA in the presence of forskolin on contractile parameters in left and right mouse atrium in the presence or absence of cantharidin. **A** Original recording of the effect of R-PIA (1 µM) in the presence of forskolin (1 µM) on the force of contraction in milli Newton (mN, Ordinate) over time in minutes (min, Abscissa). **B** Original recording of the effect of R-PIA (1 µM) in the presence of forskolin (1 µM) on the force of contraction in the additional presence of cantharidin (30 µM) in milli Newton (mN, Ordinate) over time in minutes (min, Abscissa). **C** Line diagram of the negative inotropic effect of R-PIA (1 µM) in the presence of forskolin (1 µM) or with first applied cantharidin (30 µM) in isolated electrically driven left atrial preparations. Ordinate and abscissa give the force of contraction in % of pre-drug value or applied concentration of R-PIA (1 µM), respectively. The asterisk (*) and number sign (#) indicate the first significant difference versus 30 µM cantharidin or time-matched control values (Ctr2) or R-PIA (1 µM) in the presence of cantharidin, respectively. Number (*n*) indicates the number of experiments. Ctr1 indicates pre-drug value. Ctr2 (100%) indicates plus/minus cantharidin. **D** Line diagram of the effects of R-PIA (1 µM) alone or in additional presence of cantharidin (30 µM) on the rate of tension development in isolated electrically driven mouse left atrial preparations. Ordinate and abscissa give the rate of tension development (dF/dt_max_) in % of pre-drug value or applied concentration of R-PIA, respectively. The asterisk (*) and number sign (#) indicate the first significant difference versus 30 µM cantharidin or time-matched control values (Ctr2) or R-PIA in the presence of cantharidin, respectively. Number (*n*) indicates the number of experiments. Ctr1 indicates pre-drug value. Ctr2 (100%) indicates plus/minus cantharidin. **E** Line diagram of the effects of R-PIA (1 µM) in the presence of forskolin (1 µM) or in additional presence of cantharidin (30 µM) on rate of tension relaxation in isolated electrically driven human right atrial preparations. Ordinate and abscissa give the rate of tension relaxation (dF/dt_min_) in % of pre-drug value or applied concentration of R-PIA, respectively. The asterisk (*) and number sign (#) indicate the first significant difference versus 30 µM cantharidin or time-matched control values (Ctr2) or R-PIA in the presence of cantharidin, respectively. Number (*n*) indicates the number of experiments. Ctr1 indicates pre-drug value. Ctr2 (100%) indicates plus/minus cantharidin

First, 1 µM forskolin was given to increase FOC. This concentration of forskolin stems from our previous studies in the human heart (Neumann et al. 1999a) and was also used by others in the mouse ventricular preparations to increase FOC (Wegener et al. 2002). R-PIA (1 µM based on our previous work, Schwarz et al. 2024) was applied and reduced force, as noted before in perfused whole guinea pig hearts (Baumann et al. 1981), in the absence of cantharidin after addition of forskolin (original tracing: Fig. 3A) or in the presence of cantharidin (Fig. 3B). In the presence of cantharidin, the negative inotropic effect (after forskolin had increased FOC) of R-PIA is attenuated and slower (original recordings in Fig. 3A and B). These data are summarized for the force of contraction in Fig. 3C. Note that Ctr1 indicates the developed tension in the absence of solvent or R-PIA (Fig. 3C). We define Ctr2 as the force noticed after 30 min of initial incubation with 30 µM cantharidin or solvent control and rolipram just before R-PIA was added to the organ bath. Under these conditions, in the presence of rolipram, R-PIA exerted an NIE that was larger in the absence than in the presence of cantharidin R-PIA (Fig. 3C). More results were noted for additional muscle parameters in the presence of cantharidin and forskolin. When calculating the first derivate of force versus time, one notices that the rate of tension development was enhanced by cantharidin and rolipram, but additional R-PIA was more efficient to reduce this parameter in the absence than in the presence of cantharidin (Fig. 3D). Likewise, the rate of tension relaxation was enhanced by cantharidin in the presence of rolipram, but additional R-PIA was more efficient to reduce this parameter in the absence than in the presence of cantharidin (Fig. 3E).

### Human right atrial preparations: cilostamide

**Fig. 4:**
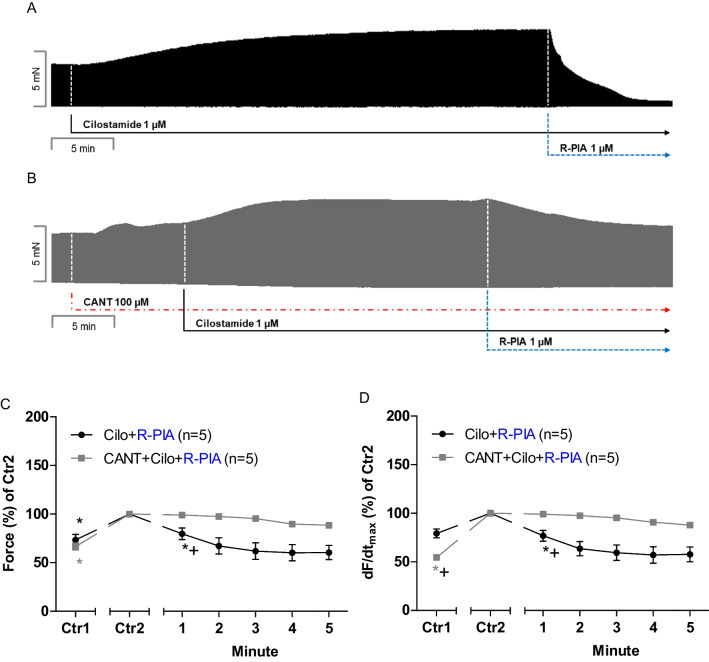
Cantharidin attenuates the negative inotropic effect of R-PIA of cilostamide on contractile parameters in the human right atrium. **A** Original recording of the effect of R-PIA (1 µM) and 1 µM cilostamide on the force of contraction in milli Newton (mN, Ordinate) over time in minutes (min, Abscissa). **B** Original recording of the effect of R-PIA and 1 µM cilostamide on the force of contraction in the presence of 100 µM cantharidin (CANT) in milli Newton (mN, Ordinate) over time in minutes (min, Abscissa). **C** Line diagram of the negative inotropic effect of R-PIA and 1 µM cilostamide or after first applied cantharidin (100 µM) in isolated electrically driven mouse left atrial preparations. Ordinates and abscissa give the force of contraction in % of pre-drug value or applied concentration of R-PIA, respectively. The asterisk (*) and plus sign ( +) indicate the first significant difference versus 100 µM cantharidin or time-matched control values (Ctr2) or R-PIA in the presence of cantharidin, respectively. Number (*n*) indicates the number of experiments. Ctr1 indicates pre-drug value. Ctr2 (100%) indicates plus/minus cantharidin. **D** Diagram of the effects of R-PIA in the presence and 1 µM cilostamide or in additional presence of cantharidin (100 µM) on rate of tension development in isolated electrically driven mouse left atrial preparations. Ordinate and abscissa give the rate of tension development (dF/dt_max_) in % of pre-drug value or applied concentration of R-PIA, respectively. The asterisk (*) and plus sign ( +) indicate the first significant difference versus 100 µM cantharidin or time-matched control values (Ctr2) or R-PIA in the presence of cantharidin, respectively. Number (*n*) indicates the number of experiments. Ctr1 indicates pre-drug value. Ctr2 (100%) indicates plus/minus cantharidin

The same protocol was used in human atrial preparations as in mice. The only difference was that we needed higher concentrations of cantharidin (100 µM) than in the mouse, as we reported before (Schwarz et al. 2024). In control experiments, the first cilostamide was given and increased FOC. When the PIE of cilostamide had reached a maximum, R-PIA was additionally applied. The R-PIA exerted an NIE in HAP. In separate muscle strips, the first cantharidin (100 µM) was utilized, this concentration of cantharidin increased FOC. Thereafter, cilostamide was given and increased FOC further. When we had reached a new plateau for FOC, then R-PIA was additionally applied. R-PIA elicited a pronounced negative inotropic effect in the presence of cilostamide (Fig. 4A). However, in the presence of cantharidin and of cilostamide, the negative inotropic effect of R-PIA is attenuated and slower to develop (original recording in Fig. 4B). These data are summarized for the negative inotropic effects in the presence of cilostamide (Fig. 4C). Comparing Ctr1 and Ctr2 in Fig. 4C, one detects the PIE of cantharidin in the presence of cilostamide. Under these conditions, cantharidin increased the rate of tension development: compare Ctr1 and Ctr2 in Fig. 4D. In the presence of cilostamide, the reductions by R-PIA of the rate of tension development were attenuated by cantharidin (Fig. 4D).

### Human right atrial preparations: forskolin

**Fig. 5:**
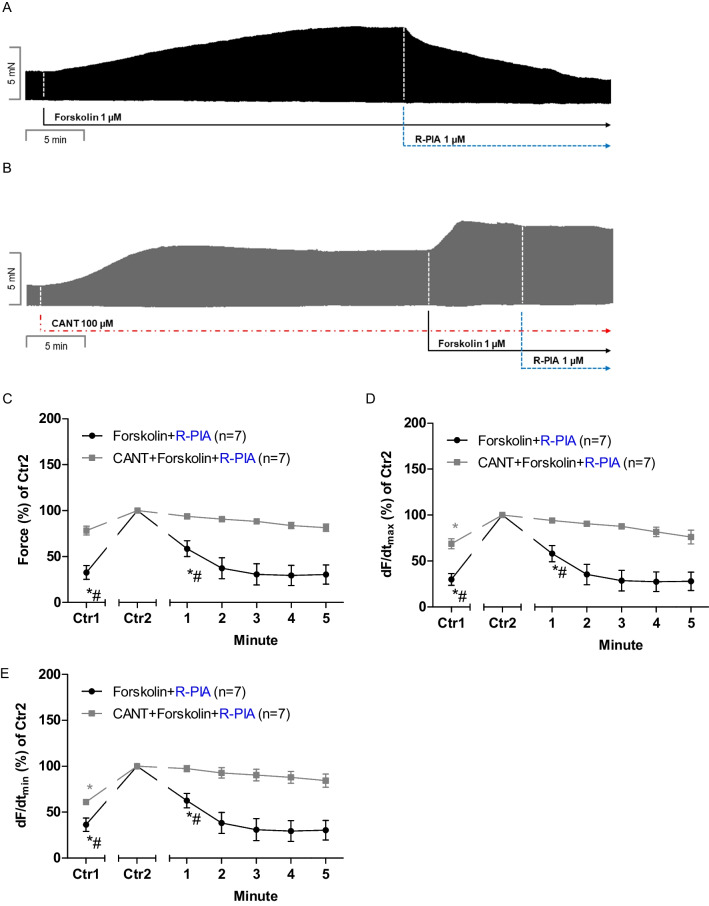
Cantharidin does not attenuate the negative inotropic effect of R-PIA in the presence of forskolin on contractile parameters in the human right atrium. **A** Original recording of the effect of R-PIA (1 µM) in the presence of forskolin (1 µM) on the force of contraction in milli Newton (mN, Ordinate) over time in minutes (min, Abscissa). **B** Original recording of the effect of R-PIA (1 µM) in the presence of forskolin (1 µM) on the force of contraction in the additional presence of cantharidin (100 µM) in milli Newton (mN, Ordinate) over time in minutes (min, Abscissa). **C** Line diagram of the negative inotropic effect of R-PIA (1 µM) in the presence of forskolin (1 µM) or with first applied cantharidin (100 µM) in isolated electrically driven human right atrial preparations. Ordinate and abscissa give the force of contraction in % of pre-drug value or applied concentration of R-PIA, respectively. The asterisk (*) and number sign (#) indicate the first significant difference versus 100 µM cantharidin or time-matched control values (Ctr2) or R-PIA in the presence of cantharidin, respectively. Number (*n*) indicates the number of experiments. Ctr1 indicates pre-drug value. Ctr2 (100%) indicates plus/minus cantharidin. **D** Line diagram of the effects of R-PIA (1 µM) in the presence of forskolin (1 µM) or in additional presence of cantharidin (100 µM) on rate of tension development in isolated electrically driven human right atrial preparations. Ordinate and abscissa give the rate of tension development (dF/dt_max_) in % of pre-drug value or applied concentration of R-PIA, respectively. The asterisk (*) and number sign (#) indicate the first significant difference versus 100 µM cantharidin or time-matched control values (Ctr2) or R-PIA in the presence of cantharidin, respectively. Number (*n*) indicates the number of experiments. Ctr1 indicates pre-drug value. Ctr2 (100%) indicates plus/minus cantharidin. **E** Line diagram of the effects of R-PIA (1 µM) in the presence of forskolin (1 µM) or in additional presence of cantharidin (100 µM) on rate of tension relaxation in isolated electrically driven human right atrial preparations. Ordinate and abscissa give the rate of tension relaxation (dF/dt_min_) in % of pre-drug value or applied concentration of R-PIA, respectively. The asterisk (*) and number sign (#) indicate the first significant difference versus 100 µM cantharidin or time-matched control values (Ctr2) or R-PIA in the presence of cantharidin, respectively. Number (*n*) indicates the number of experiments. Ctr1 indicates pre-drug value. Ctr2 (100%) indicates plus/minus cantharidin

Forskolin was given in the organ bath and increased FOC. The choice of the concentration of forskolin (1 µM) was based on previous work by us and others (human ventricle: Bristow et al. 1984, human atrium: Neumann et al. 1996, Christ et al. 2014). R-PIA elicited a pronounced negative inotropic effect in the presence of forskolin (Fig. 5A). However, in the combined presence of cantharidin and of forskolin, the NIE of R-PIA is attenuated (comparing original recordings in Fig. 5A and B). These data are summarized for the NIE of R-PIA in the presence of forskolin (Fig. 5C). Comparing Ctr1 and Ctr2 in Fig. 5C, one detects the PIE of cantharidin in the presence of forskolin. Under these conditions, cantharidin increased the rate of tension development: compare Ctr1 and Ctr2 in Fig. 5D. In the presence of forskolin, the reductions by R-PIA of the rate of tension relaxation were attenuated by cantharidin (Fig. 5E).

## Discussion

The main new findings in this report are that cantharidin attenuates the negative inotropic effect of R-PIA in the presence of cilostamide and forskolin in the human atrium.

An NIE of R-PIA via A_1_-adenosine alone or in the presence of isoprenaline, a cAMP-increasing drug, in the mouse atrium has been reported before (Neumann et al. 1999b). Adenosine inhibited the forskolin-stimulated adenylyl cyclase activity in the guinea pig ventricle (Bristow et al. 1984) and the forskolin-stimulated current through LTCC in guinea pig ventricular cardiomyocytes (Belevych et al. 2001).

We have presented evidence that isoprenaline can phosphorylate and thus activate PP-inhibitory 1 in the ventricle and thus inhibit PP1 activity (Neumann et al. 1991; Gupta et al. 1998). It is likely, but as far as we know not published, that forskolin and rolipram or cilostamide in the mammalian heart can inhibit phosphatase via the same mechanisms used for isoprenaline (Ahmad et al. 1989, Neumann et al. 1991; Gupta et al. 1998).

In guinea pig and human ventricular preparations, in contrast to atrial preparations, R-PIA alone does not decrease the force of contraction (Burnstock 2017). It is a novel finding that the PIE of cilostamide and forskolin are attenuated by R-PIA in the HAP. This is plausible if one assumes that R-PIA will reduce any effect mediated by an increase in cAMP. However, our findings are in apparent contrast to the general view that R-PIA inhibits the activity of receptor-stimulated adenylyl cyclase and, by this mechanism, reduces cAMP and thereby the force of contraction. If this were the case, then it is not easy to understand why R-PIA can reduce FOC that was elevated by impeding the degradation of cAMP. Our data in HAP are in addition are strengthened by our findings in mouse atrium: in LA, we find that the PIE of rolipram (cilostamide is inactive in mice: Neumann et al. 2019) is attenuated by R-PIA and this NIE is weakened by cantharidin. These data are supported by a recent study wherein we find the NIE of R-PIA is more pronounced in the LA of transgenic mice that overexpress in the heart the catalytic subunit of PP2A (Gergs et al. 2024).

For the interpretation of our data with forskolin, we would argue in a similar fashion. One might erroneously argue that R-PIA reduced forskolin-stimulated increases in FOC in HAP by reducing the production of cAMP. This is not the case for carbachol which acts in many respects similar to R-PIA. Likewise, a reduction in cAMP cannot explain our findings that the NIE of R-PIA in the presence of forskolin are attenuated by cantharidin. If R-PIA only acted by inhibition of the cAMP production, why should a phosphatase inhibitor interfere with this effect? In rat ventricular cardiomyocytes, forskolin increased phospholamban phosphorylation and this increase in phosphorylation was attenuated by additionally applied carbachol or R-PIA (George et al. 1991). We argue here further that our findings in HAP are supported by our findings in LA: in LA, forskolin raised FOC, and this increase was reversed by R-PIA. This NIE of R-PIA was weakened by preincubation with cantharidin. We might speculate that activation of PP by R-PIA is a general mechanism used in the mammalian heart.

There is precedence that R-PIA can activate PP1 and/or PP2A that was previously inhibited by isoprenaline in the mammalian heart. We postulate now that the same occurs in the human atrium with other cAMP dependent agents like cilostamide and forskolin. R-PIA tries to activate PP1 and/or PP2A in the human atrium but this is impaired by cantharidin. We suggest that the remaining negative inotropic effect of R-PIA in the permanent presence of cantharidin is due to activation of potassium ion or inhibition of calcium ion channels in the atrium, in a phosphorylation-independent fashion. However, there is also evidence that in HAP activation of potassium channels does not play a role for the NIE of carbachol and by extension also not for the NIE of R-PIA (Petersen et al. 2022). Cantharidin inhibits PP2A or PP1 in the guinea pig heart with IC50 values of 0.13 and 2.7 µM, respectively (Neumann et al. 1995b). This potency and selectivity is much lower than that of okadaic acid (0.7 and 120 nM, respectively, Neumann et al. 1995b). However, the concentration of cantharidin we used (30–100 µM) should have inhibited both PP1 and PP2A.

### Limitations of the study

It would be important to study cantharidin, forskolin, cilostamide, and R-PIA in human sinus node cells. At least forskolin could activate currents in human sinoatrial node-like cells (Hoekstra et al. 2021). We do not know which PP is stimulated by R-PIA in HAP because cantharidin is unspecific and how this PP is exactly stimulated by R-PIA in the human atrium. We have not studied the human ventricle. We do not know to what extent the opening of potassium channels contributes to the NIE of R-PIA in the presence of cilostamide or forskolin. We cannot exclude the possibility that A_1_ adenosine receptor activation can only reduce moderate increases in cAMP and not drastic ones like those induced by forskolin. For instance, equieffective concentrations (the same increase in FOC in human ventricular muscle strips) of forskolin (30 µM) increased cAMP levels by about 1600% (16-fold) whereas 0.2 µM isoprenaline only increase by 75% (Neumann et al. 1999a). This is also suggestive that forskolin increases cAMP in a compartment of known function. Finally, cantharidin may have additional biochemical effects in addition to phosphatase inhibition (Knapp et al. 1998). However, cantharidin increases not only protein phosphorylation in the guinea pig and human heart but also increases calcium ion transient in guinea pig ventricular cardiomyocytes suggesting an action on cardiac cells (Neumann et al. 1995b; Knapp et al. 1998; Schwarz et al. 2023a).

In summary, we can now answer the hypotheses put forward in the Introduction in the following way. Cantharidin attenuates the negative inotropic effects of R-PIA in after stimulation by cilostamide or forskolin receptors in HAP. We speculate that cantharidin might inhibit human atrial phosphatases that are stimulated by R-PIA in the presence of any cAMP-elevating compounds.

## Data Availability

All source data for this work (or generated in this study) are available upon reasonable request.
